# Neural mechanisms of motivated forgetting

**DOI:** 10.1016/j.tics.2014.03.002

**Published:** 2014-06

**Authors:** Michael C. Anderson, Simon Hanslmayr

**Affiliations:** 1 MRC Cognition and Brain Sciences Unit, University of Cambridge, Cambridge, UK; 2 Behavioural and Clinical Neuroscience Institute, University of Cambridge, Cambridge, UK; 3 School of Psychology, University of Birmingham, Birmingham, UK; 4 Department of Psychology – Zukunftskolleg, University of Konstanz, Konstanz, Germany

## Abstract

Not all memories are equally welcome in awareness. People limit the time they spend thinking about unpleasant experiences, a process that begins during encoding, but that continues when cues later remind someone of the memory. Here, we review the emerging behavioural and neuroimaging evidence that suppressing awareness of an unwelcome memory, at encoding or retrieval, is achieved by inhibitory control processes mediated by the lateral prefrontal cortex. These mechanisms interact with neural structures that represent experiences in memory, disrupting traces that support retention. Thus, mechanisms engaged to regulate momentary awareness introduce lasting biases in which experiences remain accessible. We argue that theories of forgetting that neglect the motivated control of awareness omit a powerful force shaping the retention of our past.

## Highlights

• Motivated forgetting of unwanted memories shapes what we retain of our personal past.
• Motivated forgetting is achieved in part by inhibitory control over encoding or retrieval.
• Prefrontal cortex reduces hippocampal and cortical activity to suppress memories.
• Electrophysiological activity during motivated forgetting implicates active inhibition.
• A neurobiological model of memory control can inform disordered control over memory.

## A neglected force that shapes retention

Over the past century, memory research has focused on passive factors that make us forget. Forgetting has been proposed to result from the decay of memories over time, the accumulation of similar interfering experiences in memory, and changes in physical context that make it harder to recall the past [1]. This historical emphasis on passive factors fits the common assumption that forgetting is a negative outcome and, thus, any process underlying it must happen involuntarily. Although forgetting is often negative, this emphasis neglects a fundamental feature of human existence: not all experiences are pleasant. When reminded of negative events, we are not well disposed towards them and we deliberately limit their tenure in awareness. This process is familiar to most people; a reminder evokes a brief flash of memory and feeling, abruptly followed by efforts to exclude the unwanted memory from awareness. We do this to preserve our emotional state, to protect our sense of self, and sometimes simply to concentrate on what needs to be done. Therefore, any scientific theory of forgetting must include an account of the considerable motivational forces that shape retention.

Here, we review the growing research on neural mechanisms underlying motivated forgetting. The term ‘motivated forgetting’ here refers to increased forgetting arising from active processes that down-prioritise unwanted experiences in service of creating or sustaining an emotional or cognitive state. For example, to sustain positive emotions or concentration, belief in some state of affairs, confidence, or optimism, it may be necessary to reduce accessibility of experiences that undermine those states. Here, we focus on neural evidence for the role of inhibitory control processes in the voluntary interruption of mnemonic processing. A core claim is that these inhibitory control processes, widely studied in psychology and cognitive neuroscience, can be targeted flexibly at different stages of mnemonic processing and at different types of representation to modulate the state of traces in memory.

In support of this view, we review evidence that inhibition can be engaged either during memory encoding or retrieval to limit retention of unwanted memories. Stopping encoding may disrupt the consolidation of traces already formed, and also prevent further reflection on the experience that would enhance its longevity. By contrast, stopping retrieval disrupts the automatic progression from cues to an associated memory, the persisting effects of which influence whether the experience remains accessible. Both encoding and retrieval stopping terminate an unfolding mnemonic process so that an experience can be excluded from conscious awareness. Through these efforts to terminate awareness, attentional control interacts with traces in episodic memory to shape what we do and do not remember of our past.

## Inhibitory control at encoding

An effective way of keeping an unwanted memory from being retrieved in the future is to disrupt and truncate its encoding. These processes are investigated with directed forgetting paradigms, in which participants receive a cue to forget information that they just acquired [2]. Hundreds of studies conducted over the past 50 years reveal that humans can readily implement such forgetting instructions, demonstrating that motivation indeed shapes encoding. Inhibition has been proposed to have a role in stopping encoding processes in these procedures, although passive factors also are likely to have a role (e.g., [3]). We focus here on evidence indicating a distinct contribution of inhibitory control in actively limiting the encoding of unwanted experience. This evidence has been collected with the item-method [4] and list-method [5] directed forgetting procedures (Box 1).

### Item-method directed forgetting

Item-method directed forgetting has a long tradition in cognitive psychology [4]. This effect is robust, as reflected by the range of conditions under which it has been reported, including both explicit and implicit memory tests [6,7]. Item-method directed forgetting usually has been explained in terms of selective rehearsal (see Glossary) according to which to-be-forgotten items are spared from further processing and are subject to passive forgetting, whereas to-be-remembered items are actively rehearsed [2]. Interestingly, the occurrence of item-method directed forgetting in recognition tests has been used as an argument for passive, noninhibitory explanations, because some have argued that inhibition should only temporarily reduce the accessibility of the affected items and, therefore, it should be possible to release these items from inhibition later [2].

Although selective rehearsal is a common interpretation of item-method directed forgetting [2], recent behavioural and neural evidence indicates that inhibitory control over episodic encoding may have a bigger role than has been acknowledged. For example, the selective rehearsal account emphasises processes acting on to-be-remembered items, which are rehearsed more extensively and elaborately when the cue to remember is given. Therefore, the system should experience more cognitive load in the remember compared to the ‘forget’ condition, in which people can simply drop the to-be-forgotten item from working memory. This prediction was tested in several experiments in which participants performed a secondary task after the remember and/or forget cue was given [8,9]. However, contrary to the selective rehearsal account, the forget condition was more effortful than the remember condition, as reflected by slower reaction times to perform the secondary task during execution of the forget instruction. Moreover, stopping a motor response after the cue is more successful in the forget compared with the remember condition [9], suggesting that forget cues trigger similar inhibitory mechanisms to those engaged when stopping a motor action [10]. However, further clarification of this possibility is needed [9]. These results clearly imply that an active process contributes to item-method directed forgetting [11], and raise the possibility that it is inhibitory in nature. This possibility is consistent with evidence that directed forgetting cues lead to the removal of items from working memory and not merely to passive decay [12,13].

**Figure 1:**
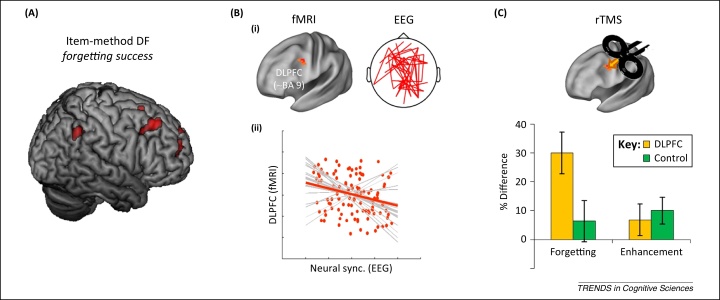
Neural correlates of directed forgetting (DF). **(A)** An activation map of a recent item-method directed forgetting functional (f)MRI study [18]. Red areas illustrate significant voxels (*P* <0.005) indicating greater activity for to-be-forgotten items that are actually forgotten compared with to-be-remembered items that are remembered. **(B,C)** The results of a multimodal list-method DF experiment [32]. (B) Forget instructions were associated with increased blood oxygenation level-dependent (BOLD) signal in the left dorsolateral prefrontal cortex (DLPFC) and reduced alpha/beta long-range synchrony [11–18 Hz (i)], which were negatively correlated on a single trial level (ii). (C) Stimulating the DLPFC with repetitive transcranial magnetic stimulation (rTMS; 1 Hz) selectively increased list-1 forgetting, without affecting list-2 enhancement. Adapted, with permission, from [32] (B,C). Abbreviation: EEG, electroencephalogram.

Several recent functional (f)MRI studies support the hypothesis that item-method directed forgetting engages an active process that inhibits ongoing encoding [14–18]. These studies consistently indicate that attempting to forget a recent item engages prefrontal and parietal regions, suggesting that forgetting is effortful, consistent with behavioural findings [15,16,18]. The right superior and middle frontal gyrus (approximately BA 9/10), and the right inferior parietal lobe (approximately BA 40) are consistently more active during intentional forgetting (to-be-forgotten items that are actually forgotten) compared with incidental forgetting (to-be-remembered items that are forgotten (Figure 1A) [15,16,18]. Although these findings suggest that intentional forgetting recruits additional processes beyond those associated with incidental forgetting, these activations do not specify the nature of those processes. For example, activations during forget trials might reflect engagement of the default mode network, which is characterised by positive blood oxygenation level-dependent (BOLD) correlations between superior prefrontal and parietal cortex during rest [19]. Thus, these findings may simply reflect a greater incidence of passive rest during forget trials compared with remember trials. However, speaking against this view, connectivity analyses show that activity in the right dorsolateral prefrontal cortex (DLPFC) during forget trials predicts decreased activity in the left hippocampus, especially during successful intentional forgetting [18]. This latter result is incompatible with the default mode network hypothesis, which predicts the opposite (positive) connectivity pattern between DLPFC and the medial temporal lobe (MTL) [20]. Rather, negative connectivity between right DLPFC and hippocampus suggests that the right prefrontal cortex exerts inhibitory control over the encoding activity in the MTL [21], similar to that observed during retrieval (see ‘Neural Basis of Retrieval Suppression’). One plausible hypothesis is that the active forgetting mechanism implicated by behavioural studies [15,16,18] may reflect the action of this frontohippocampal modulatory system.

The neural correlates of item-method directed forgetting have also been studied with intracranial event-related potentials, providing information about the temporal dynamics of the forgetting mechanism within the MTL [22]. This study found that forget cues that cause later forgetting elicited decreased negativity in the anterior hippocampus compared with remember cues that led to forgetting. Notably, enhanced negativity in the hippocampus at approximately 500 ms is usually related to successful encoding. These authors further found that forget cues triggered sustained positivity in the rhinal cortex, an interfacing structure between the cortex and hippocampus. Together with scalp event-related potential (ERP) studies, showing sustained prefrontal positivity after the forget cue [23], localised to the right DLPFC [24], these studies converge with fMRI data to suggest that item-method directed forgetting recruits a right prefrontal–MTL network to terminate episodic encoding processes. Together, these studies question a purely passive based view of item-method directed forgetting, which has been the popular account among experimental psychologists.

### List-method directed forgetting

Sometimes, we may wish to forget a set of events that is extended in time (e.g., a recent doctor's visit, or dispute with an unpleasant acquaintance). This situation is modelled by the list-method of directed forgetting (Box 1). A typical experiment comprises two lists (e.g., 10–20 items in each list), with a forget or remember cue given after the first list [2,25]. After encoding the second list, a brief distracting task follows and then recall is tested. On this final test, people typically recall the first list more poorly when it is followed by a forget, compared with a remember, instruction. Interestingly, people recall the items following a forget cue better than they do items studied after a remember cue (Box1, Figure 1C) (e.g., [25]). These complementary effects are referred to as list-1 forgetting and list-2 enhancement. These effects arise on free recall, cued recall, and recognition tests, although, in the latter case, deficits are often restricted to source memory. List-method directed forgetting effects have also been observed in autobiographical memory [26,27]. Poorer recall of the to-be-forgotten list is believed to reflect reduced accessibility rather than reduced availability of the forgotten material [2,28,29]. Both active [5,30] and passive mechanisms have also been proposed for this phenomenon [3,31].

As with item-method directed forgetting, imaging research with the list method indicates that instructions to forget trigger an active process that disrupts mnemonic activity. For example, two studies examined the neurophysiological mechanisms of directed forgetting by focussing on brain oscillations [30,32]. Prior work established that memory formation is typically accompanied by increased large scale synchrony, a neural marker thought to reflect upregulated synaptic plasticity [33–35]. Strikingly, cuing people to forget a just-studied list decreased the large-scale synchrony in a widespread cortical network in the alpha/beta frequency range. Individual differences in this effect predicted forgetting of to-be-forgotten items [30], suggesting that decreasing synchrony disrupts neural processes that would improve retention. This finding was replicated in a multimodal electroencephalography (EEG)–fMRI study [32], in which it was found to be associated with increased BOLD signal in the left DLPFC (Figure 1B). Following this discovery, a combined EEG*–*repetitive transcranial magnetic stimulation (rTMS) experiment demonstrated that stimulating the left DLPFC with rTMS during a forget instruction also reduced neural synchrony, significantly increasing directed forgetting (Figure 1C). Enhanced forgetting following rTMS indicates that stimulation facilitated processes needed to implement directed forgetting, consistent with findings showing that rTMS can enhance, rather than disrupt processing (see [32] for a detailed discussion of the enhancing effects of slow rTMS on forgetting). This finding supports a causal role of frontally driven processes in inducing forgetting effects and complements work showing that prefrontal lesions disrupt list-method directed forgetting [36].

Increased activation of the DLPFC together with decreased neural synchrony suggests that an active control process contributes to directed forgetting. However, these findings do not specify that this active process necessarily engages inhibitory control. For example, prior studies have highlighted the importance of the DLPFC in task switching (reviewed in [37]). Therefore, DLPFC involvement during the forget instruction might simply reflect a voluntarily induced task switch that stops rehearsal, and not inhibition. However, this overlap between directed forgetting and task-switching activations may be driven by inhibitory processes involved during task switching, as numerous studies indicate [38]. Furthermore, in EEG studies, task switching typically induces a pronounced increase in frontoparietal theta long-range synchrony [39], in stark contrast to the decreases in alpha/beta long-range synchrony observed in list-method directed forgetting. Although these findings suggest that directed forgetting activations are unlikely to arise from task switching, the relations between the processes in the two tasks merits further exploration.

The improved recall of list-2 that accompanies the forgetting of list-1 items might suggest a single mechanism that enhances list-2 encoding by reducing interference from list-1 items [2]. However, there are reasons to doubt this. First, forgetting and enhancement are often uncorrelated (e.g., [30,40,41]). Second, list-1 forgetting often can be modulated independently of list-2 enhancement [32,42]. Third, whereas all list-1 items suffer forgetting, irrespective of their serial position, the enhancement of list-2 appears to be driven by the first few items [42]. The latter result fits electrophysiological data suggesting that the forget cue enhances subsequent encoding because it acts like a ‘reset button’ that frees cognitive resources and allows a fresh encoding start. This assumption is reflected in EEG work showing that oscillatory markers of encoding exhaustion, which gradually increase with the number of encoded items, are reset by the forget cue [42]. This latter effect is most evident in alpha oscillatory amplitude, which usually decreases during memory encoding [43,44]. Thus, forgetting and enhancement in list-method directed forgetting appear to reflect different processes that can be dissociated on a cognitive and neural level.

The list and item methods differ in the target of forgetting. Whereas the item method targets individual items, the list method typically directs people to forget a set of items defined by temporal context (i.e., ‘the previous list’). This broader targeting may be implemented by directing inhibition at representations of temporal context rather than individual items. Consistent with this, list-method directed forgetting induces a shift away from the mental context of the first list, and this context shift may make it harder to recall list-1 items. For example, a forget instruction induces forgetting effects similar to those caused by other instructions designed merely to shift mental context away from the first list, without instructing people to forget [3]. Given that similar forgetting can be induced without instructing people to forget, some have argued that directed forgetting need not reflect inhibitory control [3]. However, an alternative possibility is that directed forgetting instructions achieve context shifts in a mechanistically distinct way, by engaging inhibitory control to force a shift in context. Consistent with this possibility, directed forgetting and ‘mental context shift’ instructions appear to be mediated by different neural processes. Whereas mental context shift instructions mainly affect local alpha and theta synchrony [45,46], only directed forgetting disrupts long-range alpha/beta neural synchrony [30,32]. Combined with evidence for a causal role of prefrontal cortex in inducing these changes in both long-range synchrony and forgetting [32], these findings suggest that an active inhibitory process disrupts list-1 context in directed forgetting. However, these dissociations are based on between-study comparisons because no study has yet directly contrasted EEG synchrony patterns between directed forgetting and mental context change.

Although inhibition may typically be targeted at temporal context in list-method directed forgetting, other targets are possible. For example, recent research has examined whether directed forgetting can be targeted selectively at some, but not all, of the pre-cue information [47–51]. Three of these studies demonstrated this is possible [48–50]. For instance, one study demonstrated selectivity of directed forgetting in three experiments using visual (colours) and auditory (words spoken by a female versus male voice) material [49]. In one experiment, list-1 items were spoken either by a male or a female voice, alternating on an item-by-item basis, and the participants were able to forget items selectively based on the gender of the speaker. However, some studies failed to find selective directed forgetting [47,51]. The reasons for these discrepancies are currently unknown. If directed forgetting is selective, it suggests that the inhibitory processes target dimensions other than temporal context.

To conclude, behavioural and neurophysiological studies indicate that encoding can be disrupted or truncated by an active inhibitory control mechanism that limits the representation of an experience in long-term memory. Similar to inhibitory control in the motor system, where higher-order control regions in the prefrontal cortex suppress activity in lower-order motor regions to stop a movement [52], the prefrontal cortex targets memory-related structures in the MTL [18,22]. These processes reduce encoding activity and downregulate long-range neural synchrony [32] to disrupt the formation of unwanted memories.

## Inhibitory control at retrieval

Unwanted experiences are often stored in memory, despite efforts to limit encoding. When this happens, limiting awareness becomes a problem of controlling retrieval. Retrieval can of course be prevented by avoiding reminders, which is a common behaviour after an unpleasant event. However, when unwelcome reminders occur, people often try to exclude the unwanted memory from awareness. Stopping retrieval of an unwanted memory is known as ‘retrieval suppression’, a process that engages response override mechanisms formally similar to stopping a reflexive motor action [53,54]. Retrieval suppression is often studied with the think/no-think paradigm (TNT) [53], which mimics situations when we try to suppress unwelcome remindings (Box 2).

### Behavioural effects of retrieval suppression

The TNT procedure consistently shows that people can limit retrieval [53–55]. Two main findings support this conclusion. First, suppressing retrieval consistently abolishes the benefits of reminders on memory, as reflected in the sizeable difference in final recall between Think and No-Think items. Thus, at a minimum, suppressing retrieval reduces the facilitation that retrieved memories usually enjoy. Second, suppressing retrieval often reduces recall for No-Think items below that observed for baseline items, a phenomenon known as ‘suppression-induced forgetting’*.* Suppression-induced forgetting is especially informative because it indicates that, during retrieval suppression, reminders do not merely fail to enhance retention, but trigger processes that impair access to the unwanted memory. These findings highlight a central theme of this article: that one's disposition towards a memory affects how well it is retained. Reminders enhance retention when a person is well disposed towards a memory, but when one has motivations for excluding a memory from awareness, retrieval can be stopped, preventing the benefits of retrieval and further disrupting the memory. These symmetrical effects of reminders indicate a high level of control over the retrieval process, control that shapes accessibility.

Much is now known about suppression-induced forgetting. First, forgetting increases with the number of times a memory is suppressed [53,55–60], indicating that suppression yields cumulative effects. The forgetting effect can be further increased if participants are given time to prepare for suppression [61], indicating the importance of anticipatory processes. Suppression-induced forgetting arises with many stimuli, including word pairs, face–scene pairs [62–65], face–word pairs [58], word–object pairs [66,67], and pairs comprising words and nonsense shapes [68]. Suppression-induced forgetting has even been observed with autobiographical experiences [69–71], although suppression impairs memory for event details more than access to the event itself. Some studies have reported a lack of suppression-induced forgetting when it might otherwise be expected (see [55] for a detailed discussion with hypotheses). Forgetting effects occur whether the memory is a neutral or negatively valenced word or scene [59,60,62–65,72–79], although it remains unclear whether forgetting increases [60,62], decreases [65,80], or is unaffected [74,75] with negative, compared with neutral valence. Although few studies have examined how long forgetting lasts, one study found that a single suppression session produces forgetting that lasts at least 24 h [81], with other evidence suggesting that it may dissipate after a week [70,80]. Suppression-induced forgetting is diminished in young children [82] and older adults [56], two populations hypothesised to have deficient inhibitory control function. Interestingly, individual differences in participants’ perceptions of their ability to control unwanted thoughts in daily life predict suppression-induced forgetting of aversive scenes [77].

Suppression-induced forgetting exhibits properties consistent with a role of inhibitory control. For example, the forgetting often generalises to novel test cues. For instance, after studying ordeal–roach, if participants suppress ‘roach’ whenever they receive ‘ordeal’ as a cue, roach will be recalled more poorly, regardless of whether it is tested with ‘Ordeal’ or ‘Insect’. Thus, suppressing a memory reduces its accessibility from a variety of cues, a property known as ‘cue independence’ [53]. Cue independence indicates that the forgetting most likely reflects disruption of the suppressed trace itself rather than the particular pathway from the reminder to the trace (reviewed in [55]; see also [83]). This is usually taken as strong evidence for an inhibition process that suppresses the trace ([53], although see [84] for an alternative]. As additional support for an item-specific inhibition process, forgetting has also been found on item recognition tests for both words and abstract shapes [68,85]. Moreover, the effect even occurs on indirect priming tests, such as perceptual identification: participants who suppress retrieval of visual objects are less likely to identify correctly those objects when they are presented in visual noise [66,67]. Thus, suppression not only impairs conscious access to unwanted memories, but also affects their unconscious influence, at least on tests of object perception.

Although research on retrieval suppression usually asks people to recall suppressed items intentionally, this arguably does not reflect real-world circumstances. In most cases, people are unlikely to try to recall experiences they were motivated to suppress. A more appropriate measure of the impact of suppression in real terms would measure the tendency to retrieve the suppressed content, rather than the ability to do so [86]. For instance, how likely would people be to respond with the suppressed content on a free association test? Interestingly, on such tests, suppression effects are especially pronounced [86]. This raises the possibility that intentional recall measures underestimate the change in spontaneous retrieval patterns that arise in real life. Changes in retrieval patterns introduced by inhibition may be sustained over the long term by alternative associations that naturally arise in response to reminders. Indeed, asking people to generate alternative associations to a reminder often increases forgetting, compared with not giving specific instructions. However, as noted shortly, thought substitution is not necessary to induce forgetting, and several mechanisms contribute to suppression-induced forgetting.

### Neural basis of retrieval suppression

Similar to directed forgetting, stopping retrieval appears to be achieved, in part, by control mechanisms mediated by the prefrontal cortex. Retrieval suppression engages lateral prefrontal cortex, including DLPFC and ventrolateral prefrontal cortex (VLPFC) often in the right hemisphere [64,87–91]. These regions resemble areas involved in stopping motor actions, suggesting that suppression engages general response override mechanisms to stop retrieval (a point to which we will return). Critically, suppression is accompanied by reduced activity in brain areas linked to episodic recollection [64,87–91]. For example, suppression is associated with reduced hippocampal activity, sometimes along with other subregions of the MTL. Given that single-unit electrophysiology and functional neuroimaging have linked hippocampal activity to the presence of retrieved memories in awareness, these findings suggest that inhibitory control interrupts hippocampal retrieval processes to suppress mnemonic awareness. Consistent with this hypothesis, frontohippocampal interactions during suppression have been observed with a range of materials, including words [87–91], visual objects [67], and negatively valenced scenes [64], suggesting a domain general suppression process.

Although the foregoing pattern suggests that suppression engages the prefrontal cortex to reduce hippocampal activity, reduced activity during no-think trials (relative to think trials) might simply reflect hippocampal engagement during think trials. Thus, rather than showing that suppression terminates retrieval, less hippocampal activity may reflect a passive failure to engage retrieval during no-think trials. However, evidence has grown that inhibitory control reduces hippocampal activation. First, hippocampal activity is also reduced compared with activity during a fixation baseline condition [64,91], suggesting that reductions reflect more than an absence of positive activation. Second, DLPFC activation during no-think trials is often negatively correlated with hippocampal activity [63,64]. Indeed, the magnitude of downregulation and the correlation with DLPFC has in some studies increased over blocks of the TNT phase [64], suggesting progressively improved hippocampal regulation with practice. Third, reduced hippocampal activity predicts later forgetting of unwanted memories [64,91]. Finally, effective connectivity analyses show a top-down modulatory influence of DLPFC on the hippocampus [67,88], with negative coupling from DLPFC predicting the amount of suppression-induced forgetting [88]. Although the pathways implementing this top-down influence are unknown, some data suggest the cingulum bundle is a plausible candidate for a white matter tract that could support the frontohippocampal interactions underlying suppression [90]. Together, these findings strongly support a role of DLPFC in reducing hippocampal activity, interrupting recollection, and impairing retention. More broadly, they specify a neurobiological model of memory control that provides a framework for understanding disordered control over memory (Box 3).

#### Opposing neural mechanisms underlie direct suppression and thought substitution

Although hippocampal downregulation is a fundamental tool of retrieval suppression, other mechanisms of controlling awareness are possible. For example, people may redirect attention to other thoughts about a reminder. Such diversionary thoughts could either prevent the entrance of the memory into awareness, or replace an intruding memory. Behavioural findings indicate that asking participants to generate thought substitutes for reminders can be effective in inducing forgetting of an unwanted memory [59,72,73,81,86,88]. Clearly, however, thought substitution could not involve suppressing retrieval. Given that the substitutes themselves need to be recollected, this approach seems to require the opposite outcome sought with retrieval suppression: the upregulation of retrieval processes.

**Figure 2:**
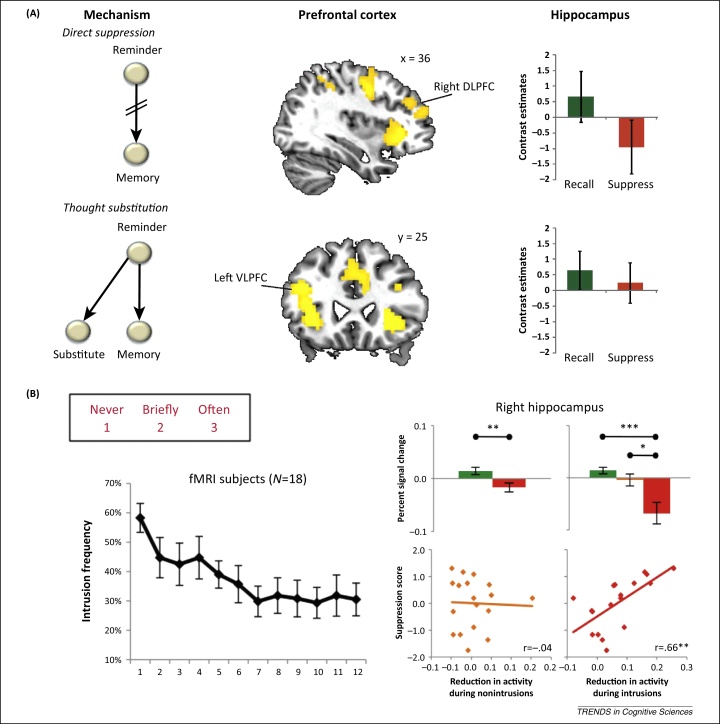
Conditions that trigger inhibitory modulation of the hippocampus during retrieval suppression. **(A)** Direct suppression and thought substitution involve distinct networks that both cause forgetting, but that have differing effects on the hippocampus [88]. Direct suppression involves suppressing episodic retrieval to prevent or override recollection of an unwanted memory (depicted by angled lines), whereas thought substitution involves engaging retrieval to recall a substitute thought in response to a reminder. Direct suppression (upper row) engages right dorsolateral prefrontal cortex (DLPFC) and ventrolateral prefrontal cortex (VLPFC), with the former reducing hippocampal activity (established by effective connectivity analyses). By contrast, thought substitution (lower row) engages a left dominant VLPFC region that does not reduce hippocampal activity (and in fact, predicts increased hippocampal activity [88]). **(B)** Measuring intrusions on no-think trials using a trial-by-trial intrusion scale (left, upper portion; ratings of 2 or 3 indicate an intrusion of the to-be-suppressed memory) reveals intrusions that decline with repeated suppressions (left lower) [91]. Strikingly, although suppression reduces hippocampal activity overall (right panel, top left subpanel; green bar, think; red bar, no-think), this modulation is driven strongly by trials on which intrusions occur (right panel, top right subpanel, red bar, intrusions; orange bar, non-intrusions). Hippocampal downregulation (pre-trial - no-think activation, z-normalized) predicts later memory deficits (baseline - no-think, z-normalized) during intrusions, but not during nonintrusions (right panel, bottom). Abbreviation: fMRI, functional MRI.

Recently, the neural mechanisms of thought substitution and inhibition in the TNT procedure have been studied [88] (Figure 2). A thought substitution group was asked to avoid unwanted memories whenever they encountered reminders to them by recalling a thought substitute to distract themselves. However, the direct suppression group was urged not to generate distracting thoughts, but to instead ‘push’ the memory from awareness, if it intruded. Interestingly, although both groups showed similar forgetting, only direct suppression reduced hippocampal activation. Critically, these strategies engaged distinct networks. Whereas direct suppression recruited the right DLPFC region typically associated with retrieval suppression, thought substitution engaged the left inferior frontal gyrus (IFG) associated with selective retrieval [92]. Effective connectivity analyses revealed that the right DLPFC was negatively coupled with the hippocampus during direct suppression, more so for people who forgot suppressed memories. By contrast, during thought substitution, activation in left caudal IFG predicted greater hippocampal activation during no-think trials, suggesting that it engaged hippocampal retrieval processes to sustain the substitute memory. Thus, two approaches to limiting awareness (suppression and self-distraction) recruited distinct frontohippocampal networks with opposing effects on hippocampal processing.

Direct suppression and thought substitution have also been dissociated electrophysiologically [93]. Prior research has established an ERP component, known as the late positive component (LPC), which is sensitive to the level of episodic recollection and contextual retrieval of a test item [94]. The LPC occurs over parietal scalp sites 400–800 ms after a recognition memory target appears, and is greater for older words than new words. If the LPC indexes recollection, measuring it during no-think trials should reveal a reduced LPC compared with that observed during think trials. This prediction has been confirmed repeatedly with word pairs [93,95–97], picture–word pairs [58] and even negatively valenced face–scene pairs [65,98]. Interestingly, participants can for the very same items, make the LPC come and go when instructions are changed from retrieval to suppression, suggesting highly efficient control over recollection [97]. Importantly, direct suppression, but not thought substitution, modulates the LPC [93]. Given that thought substitution involves recollecting substitutes (which itself would generate a LPC), the TNT conditions should be (and are) electrophysiologically similar. These findings support the view that direct suppression overrides conscious recollection, and parallel selective reductions of hippocampal activity during direct suppression [88].

#### Suppression mechanisms respond to memory intrusions

Intrusions of unwanted memories into awareness appear to have an important role in triggering inhibitory control over memory. For example, reduced hippocampal activity was closely tied to the exclusion of intrusive memories from awareness in a recent study using phenomenological reports [91]. To link intrusions to hippocampal regulation, no-think trials on which an unwanted memory entered participants’ awareness were isolated, and the intrusions were then linked to changes in hippocampal activity. Participants classified their experience after each trial according to whether the cue triggered retrieval of its associated memory. Intrusions elicited strong modulations of hippocampal activity (Figure 2). Although hippocampal downregulation occurred overall during no-think trials, the depth of reduction was pronounced during intrusions, when awareness of the memory needed to be suppressed. Strikingly, the depth of the down-regulation during intrusions strongly predicted suppression-induced forgetting. However, no correlation between downregulation and forgetting arose during nonintrusions, suggesting that reactivation of a memory trace is an important condition for memory disruption [10,99,100]. These findings link the purging of unwanted mnemonic awareness to reduced hippocampal activity. Importantly, they also show that intrusions of unwanted memories decline with repeated suppression, highlighting the outcome people seek when suppressing unwelcome remindings.

Intrusive memories seem to leap to mind automatically given reminders. The need to inhibit such automatic retrievals may engage mechanisms that are similar to those used to override reflexive actions [9,53,54]. fMRI, behavioural, and EEG evidence supports this possibility. For example, both retrieval suppression and motor inhibition engage right DLPFC and VLPFC [63,67,87–91], consistent with the similar functional demands posed by the two forms of stopping. Indeed, activation in right lateral prefrontal cortex during retrieval suppression predicts not only later retrieval suppression effects, but also stop signal reaction time on motor tasks [63]. Moreover, participants’ stop signal reaction time predicts the proportion of aversive pictures forgotten after retrieval suppression [63]. Electrophysiological components, such as the N2, are larger during suppression than during retrieval [65,85,93,95–98], echoing findings in motor inhibition research, such as the no-go N2 and the stop signal N2. Importantly, larger N2s for no-think items compared to think items are even more pronounced for no-think items that are later forgotten [96]. Strikingly, in one study, the enhanced N2 for no-think trials predicted N2 enhancement during stop-signal trials, even when the tasks were separated by a year [96]. Prior work suggests that the source of the motor no-go N2 is either the anterior cingulate cortex or the lateral prefrontal cortex [101], consistent with areas involved in retrieval suppression. Intriguingly, biomarkers of executive function know to predict individual differences in motor response inhibition, such as heart rate variability, also predict the magnitude of suppression-induced forgetting [83]. Taken together, these findings suggest that the inhibitory process engaged during retrieval suppression recruits general response inhibition mechanisms, although more precise comparison of these mechanisms is needed. For example, although memory and motor inhibition both often engage right DLPFC and VLPFC, the former has been emphasised more in research on memory inhibition (e.g., [64,87,88]), and the latter, by research on motor inhibition [52]. More work is needed to understand the roles of these two regions in these forms of stopping, and if a supramodal inhibition process exists.

#### Inhibitory control also modulates regions outside the hippocampus in a content-specific manner

**Figure 3:**
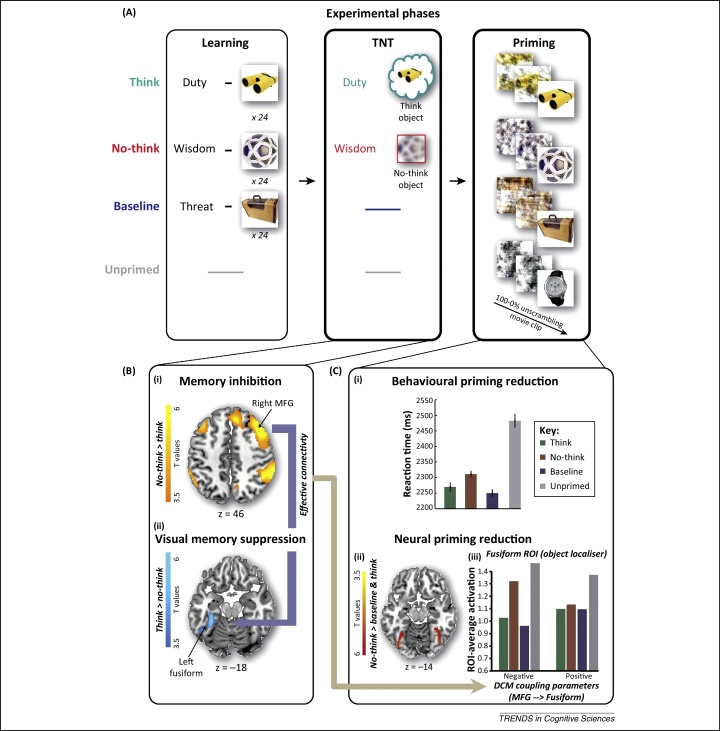
How suppressing retrieval reduces the unconscious influence of unwanted memories, via neocortical inhibition [67]. **(A)** Adaptation of the think/no-think (TNT) procedure (67). After learning word–object associations, participants either repeatedly retrieved (think) or suppressed (no-think) objects, using direct suppression [88,93]. On the final test, participants viewed objects distorted by noise that were gradually revealed, and participants indicated when they could identify the distorted object. **(B)** Suppressing retrieval activated the right dorsolateral prefrontal cortex (DLPFC) (i), and reduced activity in fusiform gyrus (ii) (effective connectivity analyses established that the former modulated the latter). **(C)** Behavioural and neural aftereffects of suppressing visual memories. All objects showed repetition priming (speeded identification time), relative to novel objects, but this was reduced for suppressed objects (i). Similarly, all studied objects showed neural priming (reduced neural activity) in fusiform gyrus and the lateral occipital complex, relative to novel objects, but this was partially reversed for suppressed objects (ii). Negative coupling between DLPFC and fusiform gyrus predicted the magnitude of the reversal in neural priming on the final perceptual identification test (iii). Abbreviations: DCM, Dynamic Causal Modelling; MGF, middle frontal gyrus; ROI, region of interest.

Although inhibitory control downregulates hippocampal activity during retrieval suppression, it also modulates activity in other brain areas, depending on the content being suppressed. For example, when people suppress retrieval of visual objects, downregulation is also observed in fusiform regions known to be critical for perceptual awareness of objects (Figure 3Bii) [67]. Interestingly, on later perceptual identification tests, participants find it more difficult to see previously suppressed objects in visual noise, compared with either baseline or think objects (Figure 3Ci), showing that motivated forgetting also impairs implicit memory [66,67]. Echoing this impaired perception, neural aftereffects are observed in the same fusiform cortex regions downregulated during retrieval suppression: no-think objects show reduced neural priming (Figure 3Cii). Given that neural priming is considered a signature of perceptual memory [102], this finding suggests that perceptual memory traces were disrupted by inhibitory control. Importantly, reductions in neural priming were well predicted by inhibitory control during the earlier TNT phase: effective connectivity analyses showed that suppressing retrieval led to negative coupling between right DLPFC and fusiform gyrus, the magnitude of which predicted the reduced neural priming in fusiform cortex on the later perception test. Thus, suppressing awareness of visual memories reduced activity not only in the hippocampus, but also in visual cortex, limiting momentary visual consciousness of the objects and disrupting later perceptual memory. This finding complements fMRI and behavioural evidence for mechanisms that purge unwanted contents from visual working memory, illustrating their inhibitory aftereffects on visual neocortex [12,103].

In the foregoing study, inhibitory control may target visual object regions to reduce reactivation arising from intrusive memories, reactivation that may arise through recurrent connections from the hippocampus [67]. This possibility suggests a broad principle of memory control: when reminders evoke activity in content-specific areas, those areas will be targeted by control [67], affecting content in those regions. Suppressing emotional memories may provide a second example. When a memory elicits a strong emotional response, regions involved in affect may be suppressed. Consistent with this, suppressing aversive scenes (e.g., violence and death) reduces activity in both the hippocampus and the amygdala ([63,64] although see [89]). Reducing activity in the amygdala could disrupt emotional learning associated with the event, much like hippocampal or fusiform modulation disrupts episodic memory or object priming, respectively. Such modulation may contribute to the widely observed fading affect bias in autobiographical memory (Box 4). However, because it remains unknown whether DLPFC is effectively connected with the amygdala during suppression, reduced activity may instead be a passive side effect of downregulating recollective activity in the hippocampus, and the resulting exclusion of the unpleasant memory from awareness. However, even given this possibility, reduced amygdala activity may reflect success at achieving a central goal of motivated forgetting in many real-life circumstances: reduced negative affect arising from the successful voluntary control of mnemonic awareness.

However, the paradigms discussed here differ from real-life circumstances in important ways. No directed forgetting or retrieval suppression paradigm, for example, captures the natural motivations that people have for suppressing awareness of memories that they find personally unwelcome (Box 4). Although the neural mechanisms identified here likely implement motivated forgetting ‘in the wild’, this work may underestimate the impact on retention for someone with a true sustained motive. Understanding the effects of personal motivation will likely entail a step away from controlled materials, towards autobiographical experiences unique to an individual. However, for now, the fundamental control processes that limit mnemonic processing are emerging, and will inform our view of how people wilfully shape retention of their personal experiences.

## Concluding remarks

In this article, we reviewed evidence for the active role that people have in shaping retention. We focussed in particular on the function of inhibitory control processes in modulating the efficacy of memory processes at both encoding and retrieval. If, upon encoding an experience, people intentionally exclude the event from awareness, retention of the experience is impaired, compared with cases in which they intend to remember the event. Although this deficit arises from several sources, one factor is the termination of encoding by inhibition, and the disruption of episodic traces formed up until that point. Similarly, upon encountering reminders to existing memories, people can engage inhibitory control to stop retrieval. In both encoding and retrieval suppression, multiple sources of evidence indicate that control mechanisms mediated by the prefrontal cortex interrupt mnemonic function and impair memory. Thus, excluding unwanted memories from awareness does not merely deprive experiences of further rehearsal, it contributes to forgetting by disrupting the suppressed memory. However, much remains to be understood about the pathways and neural mechanisms of this suppression (Box 5).

Understanding forgetting is one of the fundamental goals of the science of memory. We have argued that the focus on incidental forgetting mechanisms over the past century, although profitable, has profoundly neglected one of the most systemic forces shaping retention of life events: ourselves. Forgetting does indeed happen due to forces beyond our control; but we are, without a doubt, conspirators in our own forgetting. We wield control over mnemonic processes, choosing, among life's experiences, winners and losers for the potent effects of attention, reflection, and suppression. Modern behavioural and neurobiological research is revealing how our momentary choices to stop encoding or retrieval unfold in the brain, and how control processes disrupt the normal functioning of memory. These momentary choices are, in turn, driven by our affective, motivational, social, and cognitive goals. Thus, to understand why human beings remember what they do of their life histories, a scientific theory of forgetting must account for the foundational control mechanisms that implement the ongoing and active role that we play in shaping the fate of experience in memory.

**Glossary**

**Accessibility versus availability**: a theoretical distinction on why memory retrieval can fail. We may fail to retrieve memories because we do not access a stored memory (i.e., accessibility) or because the memory is not available anymore in the system (i.e., availability).
**Brain oscillations**: regular fluctuations visible in the EEG and/or magnetoencephalogram (MEG), most likely reflecting summated excitatory and inhibitory postsynaptic potentials. Brain oscillations occur at different distinct frequencies (up to 150 Hz) and have an important role in synchronising neural assemblies [104] and shaping synaptic plasticity [35].
**Cue independence**: the tendency for suppression-induced forgetting to generalise to novel test cues other than the one originally used as a cue during retrieval suppression.
**Direct suppression**: a method of limiting awareness of an unwanted memory when a reminder appears in which a person disengages the retrieval process to either prevent the memory for coming to mind, or to limit its time in awareness. Inhibition is thought to be a key process in overriding the natural operation of the retrieval mechanism.
**Effective connectivity analysis**: a form of connectivity analysis that allows one to infer not only that neural activity in two distinct regions is related (statistically), but also the directional nature of that relation. Effective connectivity analyses, such as dynamic causal modelling, permit causal inferences about the influence of one brain region on another in conditions of interest.
**Episodic context**: the spatiotemporal environment in which a stimulus is encountered. The representation of this context and its association to a stimulus form a fundamental feature of episodic memory of the stimulus. Context can also refer to internal states that get associated to a stimulus (e.g., mood or incidental thoughts), which is sometimes referred to as ‘mental context’.
**Event-related potential (ERP)**: a time-varying brain signal with positive and negative deflections (so-called ‘components’), obtained by averaging over several EEG segments corresponding to a task or stimulus.
**Fading affect bias**: the documented tendency for negative emotions associated with personal experiences to decline more quickly over time compared with positive emotions.
**Inhibitory control**: a control process that downregulates activity of interfering or otherwise unwanted representations in the service of a current task or goal, reducing their influence on cognition and behaviour.
**Late positive component (LPC)**: a positive ERP component related to episodic retrieval. During a retrieval task, the LPC emerges approximately 400–800 ms after stimulus onset, is maximal over parietal recording sites, and is assumed to reflect retrieval of contextual details of the study episode (i.e., recollection [105]).
**Long-range synchrony**: synchronisation between distant cell populations separated by several centimetres (e.g., frontal and parietal). Long-range synchrony is usually estimated based on the co-variation of oscillatory phase between two recording cites.
**Mnemic neglect**: the tendency for people to have a higher rate of forgetting for negative feedback about themselves and their performance, than for neutral or positive feedback, even when encoding time is matched.
**N2**: a negative ERP component related to cognitive control, and often associated with motor response inhibition. The N2 refers to enhanced frontocentral negativity typically approximately 150–400 ms.
**Repetition priming**: improved performance in processing a stimulus arising from prior exposure to the stimulus.
**Repetition suppression**: the finding that repetitions of a stimulus elicit less neural activity in areas involved in processing the stimulus, compared with nonrepeated stimuli, taken to be a marker of memory for the stimulus.
**Repetitive transcranial magnetic stimulation (rTMS)**: a technique commonly used to stimulate a specific brain area by applying a time-varying magnetic field that induces electric current flow in the brain.
**Selective rehearsal**: a passive, noninhibitory account used to explain the reduced memory performance for to-be-forgotten items, relative to to-be-remembered items.
**Socially shared retrieval-induced forgetting**: when a person is recounting an experience shared by listeners, the tendency for the listeners to later forget (at a higher rate) details not recounted by the speaker. The higher rate of forgetting is thought to arise from listeners covertly retrieving the experience as it is being recounted and, consequently, inducing retrieval-induced forgetting on nonretrieved knowledge.
**Suppression-induced forgetting**: in the TNT procedure, impaired recall of no-think items, compared with baseline memories that are neither retrieved nor suppressed.
**Think/no-think procedure (TNT)**: the main procedure used to study retrieval suppression, whereby people are repeatedly prompted with cues to memories and asked to either retrieve (think) the memory, or to stop its retrieval (no-think), with the result that suppressed items are more poorly recalled on later tests.
**Thought substitution**: a method of preventing retrieval of an unwanted memory when a reminder appears in which a person generates alternative thoughts associated to the reminder to occupy awareness.

## Figures and Tables

**Figure I:**
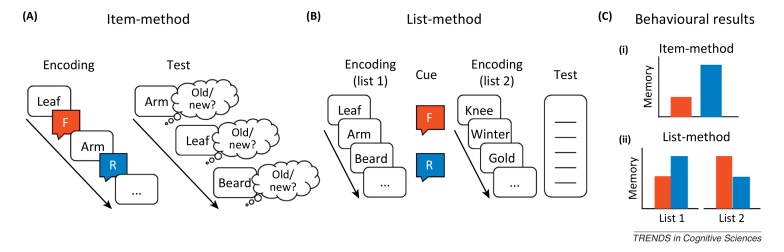
The item and list-methods for studying directed forgetting, along with the typical pattern of findings (for real examples, see [8] and [5,25], respectively).

**Figure I:**
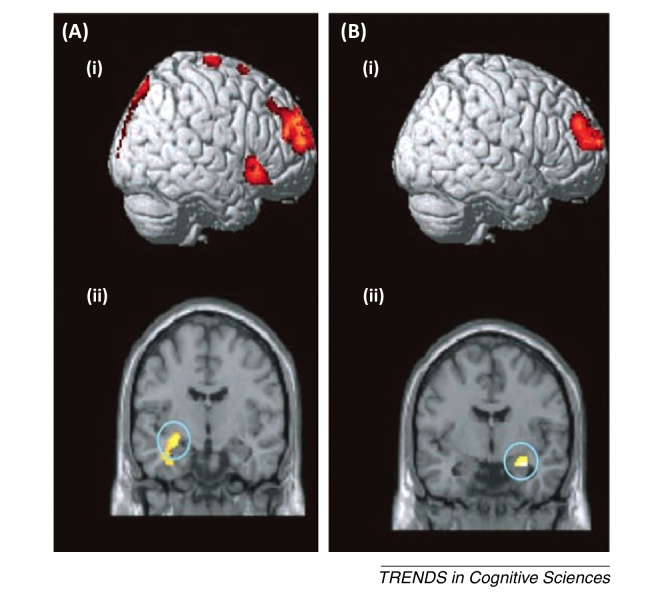
Brain-imaging data from two patients with dissociative amnesia [116]. Patients 1 **(A)** and 2 **(B)** viewed images of faces and decided whether they recognised them from their life. Images were either strangers (novel), faces they knew, from outside the window of amnesia (identifiable faces) or faces they knew from within the amnesic window (unidentifiable faces). (Ai) and (Bi) depict brain areas that are more active for unidentifiable faces than for identifiable faces (right dorsolateral prefrontal cortex). (Aii) and (Bii) depict brain areas that are less active for unidentifiable faces (hippocampus).

## Box 1 Item and list-method directed forgetting

In studies of directed forgetting, two procedures are generally used: the item-method and the list-method (see [1]). These paradigms are illustrated in Figure I. As illustrated in Figure IA, in item-method directed forgetting, participants study items one at a time, and each item is followed by a forget (F) or remember (R) instruction. Later, memory for all items is tested. As shown in Figure IB, in list-method directed forgetting, a entire list of items is first studied, followed by a F or R instruction. A second list is then studied, usually followed by a R instruction. At the end, a recall test occurs. Figure IC compares the typical behavioural results obtained in item-method (Figure ICi) and list-method (Figure ICii) directed forgetting. Figure ICii shows the twofold effect of the forget cue on the recall test, with forgetting of list-1 items and enhancement of list-2 items. In both paradigms, participants do not know in advance whether they should forget or remember the respective item. Thus, the control processes mediating these effects must act on memory representations and not the initial perception of an event.

## Box 2 The TNT paradigm

The TNT procedure mimics situations in which we encounter a reminder to a memory we prefer not to think about, and try to keep the memory out of mind [53]. To create reminders, participants study cue–target pairs (e.g., word pairs, or picture pairs, such as ‘ordeal roach’) and are then trained to recall the second item (roach) of the pair whenever they encounter the first (ordeal) as a reminder. Participants then participate in the TNT phase, in which they are asked to exert control over retrieval. On each trial, reminders from the pairs appear in green or red; when the reminder appears in green, participants are to recall the response, whereas, for red reminders, participants are asked to avoid retrieving the response, preventing it from entering awareness. The key question concerns whether people can recruit inhibition to prevent the memory from intruding into consciousness, and whether doing so disrupts later retention of the unwanted memory.

To measure the effects of retrieval suppression, participants receive a final test in which they are given each reminder and are asked to recall the associated response. Memory performance is compared between items that participants suppressed (no-think trials), items that they retrieved (think trials), and items that they studied, but neither suppressed nor retrieved during the TNT phase (baseline trials). Comparing final recall of no-think items to either think or baseline items indicates whether retrieval suppression affects retention. Comparing no-think to baseline items is more appropriate when trying to establish that suppression makes memory worse, as opposed to merely preventing memory improvement that might arise from repeated reminders.

## Box 3 Clinical variation in motivated forgetting

Individual differences in memory control may cause either deficient or exaggerated rates of forgetting of life events [55,106,107] that we might better understand and remediate with a neurobiological model of memory control.

### Deficient memory control

Intrusive memories and thoughts arise in many clinical conditions, such as post-traumatic stress disorder (intrusions), depression (rumination), attention deficit disorder (distracting thoughts), obsessive/compulsive disorder (obsessive thoughts), addiction (craving related thoughts), and anxiety (worries). This symptom may originate from deficient inhibitory control over memory. Supporting this, during retrieval suppression, adults with attention deficit disorder show impaired suppression-induced forgetting, and also diminished modulation of hippocampal activity by DLPFC [63]. Similarly, patients with post-traumatic stress disorder show impaired response inhibition [108], diminished engagement of lateral prefrontal cortex on response inhibition tasks [109], and, critically, reduced directed forgetting [110]. Rumination, depression, and anxiety have also been linked to impaired suppression-induced forgetting [79,111,112].

Deficient memory control may reflect compromised function of the networks discussed in this article. For example, disordered control may originate from diminished cortical volume or white matter connectivity between prefrontal cortex and sites of modulation. Deficits in neurotransmitters relating to inhibitory control, such as dopamine, might also underpin disordered control, a possibility supported by work linking genetic variation in dopamine metabolism to memory inhibition [113]. Alternatively, poor memory control may sometimes reflect lack of experience with intrusive memories and, thus, inadequate development of the control process, which may exhibit experience dependent plasticity. For example, after a trauma, cortical thickness in right DLPFC increases significantly over a year, with the size of the increase predicting reduced post-traumatic stress disorder symptoms [114]. Taken together, these findings suggest that both pharmacological and training interventions could be designed to bolster memory control.

### Exaggerated memory control

One striking example of motivated forgetting is psychogenic amnesia, in which a person exhibits profound amnesia for large chunks of their personal experiences in the aftermath of an intensely stressful period [115]. Two studies suggest that such cases in part reflect exceptionally effective memory control. One study examined two psychogenic amnesia patients, with amnesia extending years before scanning [116]. Both patients were scanned as they identified faces. Some faces were of strangers (novel faces). Others were of people the patients knew, with half drawn from people they met before their window of amnesia (identifiable faces), and the other half from during the window of time affected by amnesia (unidentifiable faces). Unsurprisingly, patients did not recognise the novel faces, and could recognise all of the identifiable faces. Intriguingly, although neither patient remembered any of the unidentifiable faces, these faces elicited increased activation in right DLPFC and VLPFC, together with reduced activity in the hippocampus, as observed in laboratory studies of retrieval suppression (Figure I). After treatment, one patient recovered their memories and, upon rescanning, no longer exhibited the suppression pattern. These findings suggest that extreme psychological distress leads retrieval suppression to be engaged involuntarily in reaction to certain stimuli [116]. In an independent study, a patient with psychogenic amnesia was shown to exhibit dramatically magnified suppression-induced forgetting in the TNT procedure [117], suggesting a link between their condition and suppression ability.

## Box 4 Motives for motivated forgetting

Below is a sampling of motives that may trigger motivated forgetting, illustrating the breadth of contexts in which these neural mechanisms are likely to operate.

### Regulating negative affect

Memories that evoke fear, anger, sadness, guilt, shame, anxiety, and embarrassment trigger people to regulate their emotions by suppressing offending memories [118]. In the short term, emotion regulation helps to reduce negative feelings, returning to a state of homeostasis [119]. In the long term, this may contribute to the reliably reduced frequency of negative autobiographical memories compared with positive ones for most people [120,121]. It may also contribute to the fading affect bias, wherein affect associated with negative memories fades more rapidly than other affective content [120,122].

### Justifying inappropriate behaviour

People sometimes engage in dishonest acts that conflict with their desire to be moral. This dissonance creates discomfort that people may reduce via motivated forgetting. In experimental settings, people show increased forgetting of moral rules after behaving dishonestly (e.g., cheating) even though they are equally likely to remember morally irrelevant rules as participants who do not cheat [123,124].

### Maintaining beliefs and attitudes

People's beliefs are often resistant to contradictory evidence. This resilience may be supported by selectively forgetting information not congenial to one's beliefs. For example, republicans and democrats show enhanced directed forgetting for attitude statements that are incongruent with their beliefs, compared with congruent statements [125]. Moreover, one's memory can be shaped by selectively recounting elements of an event [126–128], a form of thought substitution [88,129]. Intriguingly, this can undermine listeners’ memories of the omitted facts, a phenomenon called ‘socially shared retrieval-induced forgetting’ [126–128].

### Deceiving others and oneself

Memory inhibition may contribute to creating a state of false belief, necessary to deceive others and even oneself [130]. Consistent with this, people can use retrieval suppression to disguise guilty knowledge of a crime when confronting reminders to the crime event, effectively eliminating EEG markers of recollection [131].

### Preserving self image

People protect their self-image by selectively remembering feedback consistent with positive traits and forgetting that which threatens their sense of self. This robust ‘mnemic neglect’ effect arises despite holding encoding time constant, and is only present for encoding traits in relation to oneself, not others. Mnemic neglect is markedly attenuated, if, before encoding, people receive positive, self-enhancing feedback on a separate task, reducing their urge for self-protection [132–134].

### Forgiving others

Interpersonal relations are sometimes accompanied by the need to forgive relationship partners for offenses that provoke anger. Individual differences in forgiveness are well predicted by inhibitory control ability [135], and it has been argued that memory inhibition may be key in overcoming rumination about transgressions [136]. Forgiving and forgetting may indeed be closely related.

### Maintaining attachment

The need to maintain an attachment relationship with a parent, guardian, or powerful authority figure (e.g., a boss) may be essential to survive or thrive in an environment. Behaviours that promote good relations or attachment to the influential individual may motivate selective remembering of experiences compatible with attachment [129], and forgetting of those that are incompatible. Betrayal trauma theory, for example, posits that motivated forgetting of childhood abuse by a trusted caretaker is driven by this attachment need [137,138].

## Box 5 Outstanding questions

• How do motivation and emotion influence the ability to inhibit memories?
• What are the critical pathways by which DLPFC modulates neural activity in the hippocampus or neocortex to suppress memories?
• Is there plasticity in the networks underlying memory control that might be exploited to train people's management of intrusive memories?
• What neural changes underlie the disrupted memory performance associated with memory inhibition and is it related to reconsolidation?
• Are the inhibitory control mechanisms that support the stopping of encoding and retrieval the same?
• How is activity in the prefrontal–hippocampal memory control network orchestrated by means of brain oscillations?
• Do cases of psychogenic amnesia arise from motivated forgetting mechanisms discussed here, or is psychogenic amnesia qualitatively different?

## References

[1] Baddeley A. (2009). Memory.

[2] Bjork R.A., Roediger H.L., Craik F.I.M. (1989). Retrieval inhibition as an adaptive mechanism in human memory. Varieties of Memory and Consciousness: Essays in Honour of Endel Tulving.

[3] Sahakyan L., Ross B.H. (2013). List-method directed forgetting in cognitive and clinical research: a theoretical and methodological review.

[4] Muther W.S. (1965). Erasure of partitioning in short-term memory. Psychon. Sci..

[5] Geiselman R.E.R. (1983). Disrupted retrieval in directed forgetting: a link with posthypnotic amnesia. J. Exp. Psychol. Gen..

[6] Basden B.H., Basden D.R., Golding J.M., MacLeod C.M. (1998). Directed forgetting: a contrast of methods and interpretations. Intentional forgetting: Interdisciplinary approaches.

[7] MacLeod C.M., Daniels K.A. (2000). Direct versus indirect test of memory: directed forgetting meets the generation effect. Psychon. Bull. Rev..

[8] Fawcett J.M., Taylor T.L. (2008). Forgetting is effortful: evidence from reaction time probes in an item-method directed forgetting task. Mem. Cognit..

[9] Fawcett J.M., Taylor T.L. (2010). Directed forgetting shares mechanisms with attentional withdrawal but not with stop-signal inhibition. Mem. Cognit..

[10] Anderson M.C. (2003). Rethinking interference theory: executive control and the mechanism of forgetting. J. Mem. Lang..

[11] Zacks R.T. (1996). Studies of directed forgetting in older adults. J. Exp. Psychol. Learn. Mem. Cogn..

[12] Williams M. (2013). The benefit of forgetting. Psychon. Bull. Rev..

[13] Ecker U.K.H. (2013). Removal of information from working memory: a specific updating process. J. Mem. Lang..

[14] Reber P.J. (2002). Neural correlates of successful encoding identified using functional magnetic resonance imaging. J. Neurosci..

[15] Wylie G.R. (2007). Forgetting as an active process: an fMRI investigation of item-method-directed forgetting. Cereb. Cortex.

[16] Nowicka A. (2011). Forgetting of emotional information is hard: an fMRI study of directed forgetting. Cereb. Cortex.

[17] Rauchs G. (2011). Sleep contributes to the strengthening of some memories over others, depending on hippocampal activity at learning. J. Neurosci..

[18] Rizio A.A., Dennis N.A. (2013). The neural correlates of cognitive control: successful remembering and intentional forgetting. J. Cogn. Neurosci..

[19] Raichle M.E. (2001). A default mode of brain function. Proc. Natl. Acad. Sci. U.S.A..

[20] Vincent J.L. (2008). Evidence for a frontoparietal control system revealed by intrinsic functional connectivity. J. Neurophysiol..

[21] Paller K.A., Wagner A.D. (2002). Observing the transformation of experience into memory. Trends Cogn. Sci..

[22] Ludowig E. (2010). Active suppression in the mediotemporal lobe during directed forgetting. Neurobiol. Learn. Mem..

[23] Paz-Caballero M. (2004). Predictive validity of event-related potentials (ERPs) in relation to the directed forgetting effects. Clin. Neurophysiol..

[24] Hauswald A. (2011). ERP dynamics underlying successful directed forgetting of neutral but not negative pictures. Soc. Cogn. Affect. Neurosci..

[25] Bäuml K-H. (2010). Binding and inhibition in episodic memory: cognitive, emotional, and neural processes. Neurosci. Biobehav. Rev..

[26] Barnier A. (2007). Directed forgetting of recently recalled autobiographical memories. J. Exp. Psychol. Gen..

[27] Joslyn S.L., Oakes M.A. (2005). Directed forgetting of autobiographical events. Mem. Cognit..

[28] Bäuml K.H., Samenieh A. (2010). The two faces of memory retrieval. Psychol. Sci..

[29] Bäuml K.H., Samenieh A. (2012). Selective memory retrieval can impair and improve retrieval of other memories. J. Exp. Psychol. Learn. Mem. Cogn..

[30] Bäuml K-H. (2008). Oscillatory correlates of intentional updating in episodic memory. Neuroimage.

[31] MacLeod C.M., Ross B.H. (2003). In opposition to inhibition.

[32] Hanslmayr S. (2012). Prefrontally driven downregulation of neural synchrony mediates goal-directed forgetting. J. Neurosci..

[33] Weiss S., Rappelsberger P. (2000). Long-range EEG synchronization during word encoding correlates with successful memory performance. Brain Res. Cogn. Brain Res..

[34] Fell J. (2001). Human memory formation is accompanied by rhinal-hippocampal coupling and decoupling. Nat. Neurosci..

[35] Fell J., Axmacher N. (2011). The role of phase synchronization in memory processes. Nat. Rev. Neurosci..

[36] Conway M.A., Fthenaki A. (2003). Disruption of inhibitory control of memory following lesions to the frontal and temporal lobes. Cortex.

[37] Monsell S. (2003). Task switching. Trends Cogn. Sci..

[38] Koch I. (2010). The role of inhibition in task switching: a review. Psychon. Bull. Rev..

[39] Sauseng P. (2006). Relevance of theta and alpha oscillations during task switching. Exp. Brain Res..

[40] Conway M.A. (2000). The disruption and dissolution of directed forgetting: inhibitory control of memory. J. Mem. Lang..

[41] Sahakyan L., Goodmon L.B. (2007). The influence of directional associations on directed forgetting and interference. J. Exp. Psychol. Learn. Mem. Cogn..

[42] Pastötter B., Bäuml K-H. (2010). Amount of postcue encoding predicts amount of directed forgetting. J. Exp. Psychol. Learn. Mem. Cogn..

[43] Hanslmayr S. (2012). Oscillatory power decreases and long-term memory: the information via desynchronization hypothesis. Front. Hum. Neurosci..

[44] Hanslmayr S., Staudigl T. (2013). How brain oscillations form memories: processing based perspective on oscillatory subsequent memory effects. Neuroimage.

[45] Pastötter B. (2008). Oscillatory brain activity before and after an internal context change: evidence for a reset of encoding processes. Neuroimage.

[46] Staudigl T., Hanslmayr S. (2013). Theta oscillations at encoding mediate the context-dependent nature of human episodic memory. Curr. Biol..

[47] Sahakyan L. (2004). Destructive effects of ‘forget’ instructions. Psychon. Bull. Rev..

[48] Delaney P.F. (2009). The selective directed forgetting effect: can people forget only part of a text?. Q. J. Exp. Psychol..

[49] Kliegl O. (2012). List-method directed forgetting can be selective: evidence from the 3-list and the 2-list tasks. Mem. Cognit..

[50] Gómez-Ariza C.J. (2013). Selective intentional forgetting in adolescents with social anxiety disorder. Psychiatry Res..

[51] Storm B.C. (2013). Selective cues to forget can fail to cause forgetting. Q. J. Exp. Psychol. (Colchester).

[52] Aron A.R. (2004). Inhibition and the right inferior frontal cortex. Trends Cogn. Sci..

[53] Anderson M.C., Green C.C. (2001). Suppressing unwanted memories by executive control. Nature.

[54] Anderson M.C., Levy B.J. (2009). Suppressing unwanted memories. Curr. Direct. Psychol. Sci..

[55] Anderson M.C., Huddleston E., Belli R.F. (2011). Towards a cognitive and neurobiological model of motivated forgetting. True and False Recovered Memories: Toward A Reconciliation Of The Debate.

[56] Anderson M.C. (2011). Intentional suppression of unwanted memories grows more difficult as we age. Psychol. Aging.

[57] Joormann J. (2009). Training forgetting of negative material in depression. J. Abnorm. Psychol..

[58] Hanslmayr S. (2009). Anticipatory signatures of voluntary memory suppression. J. Neurosci..

[59] Joormann J. (2005). Remembering the good, forgetting the bad: intentional forgetting of emotional material in depression. J. Abnorm. Psychol..

[60] Lambert A.J. (2010). Testing the repression hypothesis: effects of emotional valence on memory suppression in the think-no think task. Conscious. Cogn..

[61] Hanslmayr S. (2010). Anticipation boosts forgetting of voluntarily suppressed memories. Memory.

[62] Depue B.E. (2006). Suppression of emotional and nonemotional content in memory: effects of repetition on cognitive control. Psychol. Sci..

[63] Depue B.E. (2010). Inhibitory control of memory retrieval and motor processing associated with the right lateral prefrontal cortex: evidence from deficits in individuals with ADHD. Neuropsychologia.

[64] Depue B.E. (2007). Prefrontal regions orchestrate suppression of emotional memories via a two-phase process. Science.

[65] Chen C. (2012). Suppression of aversive memories associates with changes in early and late stages of neurocognitive processing. Neuropsychologia.

[66] Kim K., Yi D-J. (2013). Out of mind, out of sight: perceptual consequences of memory suppression. Psychol. Sci..

[67] Gagnepain P. (2014). Suppressing unwanted memories reduces their unconscious influence via targeted cortical inhibition. Proc. Natl. Acad. Sci. U.S.A..

[68] Hart R.E., Schooler J.W. (2012). Suppression of novel stimuli: changes in accessibility of suppressed nonverbalizable shapes. Conscious. Cogn..

[69] Noreen S., MacLeod M.D. (2013). It's all in the detail: Intentional forgetting of autobiographical memories using the autobiographical think/no–think task. J. Exp. Psychol. Learn. Mem. Cogn..

[70] Noreen S., MacLeod M.D. (2014). To think or not to think, that is the question: individual differences in suppression and rebound effects in autobiographical memory. Acta Psychol. (Amst.).

[71] Stephens E. (2013). Suppression-induced reduction in the specificity of autobiographical memories. Clin. Psychol. Sci..

[72] Hertel P., McDaniel L. (2010). The suppressive power of positive thinking: aiding suppression-induced forgetting in repressive coping. Cogn. Emot..

[73] LeMoult J., Hertel P.T. (2010). Training the forgetting of negative words: the role of direct suppression and the relation to stress reactivity. Appl. Cogn. Psychol..

[74] Murray B.D. (2011). Effects of emotion and age on performance during a think/no–think memory task. Psychol. Aging.

[75] van Schie K. (2013). Emotional and non-emotional memories are suppressible under direct suppression instructions. Cogn. Emot..

[76] Kim D.Y. (2013). Effects of intentional suppression of recall of unwanted images in repressors and non-repressors. Soc. Behav. Personal..

[77] Kuepper C. (2014). Direct suppression as a mechanism for controlling unpleasant memories in daily life. J. Exp. Psychol. Gen..

[78] Deok-Yong K. (2013). Effects of intentional suppression of recall of unwanted images in repressors and nonrepressors. Soc. Behav. Pers..

[79] Marzi T. (2014). Emotions shape memory suppression in trait anxiety. Front. Psychol..

[80] Nørby S. (2010). Forgetting to forget: on the duration of voluntary suppression of neutral and emotional memories. Acta Psychol. (Amst.).

[81] Hotta C., Kawaguchi J. (2009). Self-initiated use of thought substitution can lead to long term forgetting. Psychologia.

[82] Paz-Alonso P.M. (2009). Memory suppression is an active process that improves over childhood. Front. Hum. Neurosci..

[83] Gillie B.L. (2013). Heart rate variability predicts control over memory retrieval. Psychol. Sci..

[84] Tomlinson T.D. (2009). An interference account of cue-independent forgetting in the no-think paradigm. Proc. Natl. Acad. Sci. U.S.A..

[85] Waldhauser G. (2012). Intentional suppression can lead to a reduction of memory strength: behavioral and electrophysiological findings. Front. Psychol..

[86] Hertel P.T. (2012). Suppression-induced forgetting on a free association test. Memory.

[87] Anderson M.C. (2004). Neural systems underlying the suppression of unwanted memories. Science.

[88] Benoit R.G., Anderson M.C. (2013). Opposing mechanisms support the voluntary forgetting of unwanted memories. Neuron.

[89] Butler A.J., James K.H. (2010). The neural correlates of attempting to suppress negative versus neutral memories. Cogn. Affect. Behav. Neurosci..

[90] Paz-Alonso P.M. (2013). Strength of coupling within a mnemonic control network differentiates those who can and cannot suppress memory retrieval. J. Neurosci..

[91] Levy B.J., Anderson M.C. (2012). Purging of memories from conscious awareness tracked in the human brain. J. Neurosci..

[92] Kuhl B.A. (2007). Decreased demands on cognitive control reveal neural processing benefits of forgetting. Nat. Neurosci..

[93] Bergström Z.M. (2009). ERP and behavioural evidence for direct suppression of unwanted memories. Neuroimage.

[94] Friedman D., Johnson R. (2000). Event-related potential (ERP) studies of memory encoding and retrieval: a selective review. Microsc. Res. Tech..

[95] Bergström Z.M. (2007). ERP evidence for successful voluntary avoidance of conscious recollection. Brain Res..

[96] Mecklinger A. (2009). ERP correlates of intentional forgetting. Brain Res..

[97] Bergström Z.M. (2009). Event-related potential evidence that automatic recollection can be voluntarily avoided. J. Cogn. Neurosci..

[98] Depue B. (2013). ERPs and Neural oscillations during volitional suppression of memory retrieval. J. Cogn. Neurosci..

[99] Lee J.L. (2009). Reconsolidation: maintaining memory relevance. Trends Neurosci..

[100] Detre G.J. (2013). Moderate levels of activation lead to forgetting in the think/no-think paradigm. Neuropsychologia.

[101] Lavric A. (2004). When ‘go’ and ‘nogo’ are equally frequent: ERP components and cortical tomography. Eur. J. Neurosci..

[102] Grill-Spector K. (2006). Repetition and the brain: neural models of stimulus-specific effects. Trends Cogn. Sci..

[103] Gazzaley A. (2008). Age-related top-down suppression deficit in the early stages of cortical visual memory processing. Proc. Natl. Acad. Sci. U.S.A..

[104] Fries P. (2005). A mechanism for cognitive dynamics: neural communication through neuronal coherence. Trends Cogn. Sci..

[105] Rugg M.D., Yonelinas A.P. (2003). Human recognition memory: a cognitive neuroscience perspective. Trends Cogn. Sci..

[106] Levy B.J., Anderson M.C. (2008). Individual differences in the suppression of unwanted memories: the executive deficit hypothesis. Acta Psychol. (Amst.).

[107] Depue B.E. (2012). A neuroanatomical model of prefrontal inhibitory modulation of memory retrieval. Neurosci. Biobehav. Rev..

[108] Aupperle R.L. (2012). Executive function and PTSD: disengaging from trauma. Neuropharmacology.

[109] Falconer E. (2008). The neural networks of inhibitory control in posttraumatic stress disorder. J. Psychiatry Neurosci..

[110] Zwissler B. (2011). Memory control in post-traumatic stress disorder: evidence from item method directed forgetting in civil war victims in Northern Uganda. Psychol. Med..

[111] Hertel P.T., Gerstle M. (2003). Depressive deficits in forgetting. Psychol. Sci..

[112] Dieler A.C. (2014). Voluntary suppression of thoughts is influenced by anxious and ruminative tendencies in healthy volunteers. Memory.

[113] Wimber M. (2011). Prefrontal dopamine and the dynamic control of human long-term memory. Transl. Psychiatry.

[114] Lyoo I.K. (2011). The neurobiological role of the dorsolateral prefrontal cortex in recovery from trauma: longitudinal brain imaging study among survivors of the South Korean subway disaster. Arch. Gen. Psychiatry.

[115] Kopelman M.D. (2002). Disorders of memory. Brain.

[116] Kikuchi H. (2010). Memory repression: brain mechanisms underlying dissociative amnesia. J. Cogn. Neurosci..

[117] Tramoni E. (2009). Hypo-retrieval and hyper-suppression mechanisms in functional amnesia. Neuropsychologia.

[118] Erdelyi M.H. (2006). The unified theory of repression. Behav. Brain Sci..

[119] Taylor S.E. (1991). Asymmetrical effects of positive and negative events: the mobilization–minimization hypothesis. Psychol. Bull..

[120] Walker W.R. (2003). Life is pleasant – and memory helps to keep it that way!. Rev. Gen. Psychol..

[121] Storm B.C., Jobe T.A. (2012). Retrieval-induced forgetting predicts failure to recall negative autobiographical memories. Psychol. Sci..

[122] Walker W.R. (1997). Autobiographical memory: unpleasantness fades faster than pleasantness over time. Appl. Cognit. Psychol..

[123] Shu L.L., Gino F. (2012). Sweeping dishonesty under the rug: how unethical actions leads to forgetting of moral rules. J. Pers. Soc. Psychol..

[124] Shu L.L. (2011). Dishonest deed, clear conscience: when cheating leads to moral disengagement and motivated forgetting. Pers. Soc. Psychol. Bull..

[125] Waldum E., Sahakyan L. (2012). Putting congeniality effects into context: investigating the role of context in attitude memory using multiple paradigms. J. Mem. Lang..

[126] Cuc A. (2007). Silence is not golden: a case for socially shared retrieval-induced forgetting. Psychol. Sci..

[127] Stone C.B. (2012). Toward a science of silence: the consequences of leaving a memory unsaid. Perspect. Psychol. Sci..

[128] Stone C.B. (2012). Forgetting our personal past: socially shared retrieval-induced forgetting of autobiographical memories. J. Exp. Psychol. Gen..

[129] Anderson M.C. (2001). Active forgetting: evidence for functional inhibition as a source of memory failure. J. Aggress. Maltreat. Trauma.

[130] Von Hippel W., Trivers R. (2011). The evolution and psychology of self-deception. Behav. Brain Sci..

[131] Bergstrom Z. (2013). Intentional retrieval suppression can conceal guilty knowledge in ERP memory detection tests. Biol. Psychol..

[132] Sedikides C., Green J.D. (2009). Memory as a self-protective mechanism. Soc. Personal. Psychol. Compass.

[133] Sedikides C., Green J.D. (2004). What I don’t recall can’t hurt me: information negativity versus information inconsistency as determinants of memorial self-defense. Soc. Cognit..

[134] Sedikides C., Green J.D. (2000). On the self-protective nature of inconsistency/negativity management: using the person memory paradigm to examine self-referent memory. J. Pers. Soc. Psychol..

[135] Wilkowski B.M. (2010). How does cognitive control reduce anger and aggression? The role of conflict monitoring and forgiveness processes. J. Pers. Soc. Psychol..

[136] Pronk T. (2010). What it takes to forgive: when and why executive functioning facilitates forgiveness. J. Pers. Soc. Psychol..

[137] Freyd J.J. (1996). Betrayal Trauma: The Logic Of Forgetting Childhood Abuse.

[138] Freyd J.J. (2006). Self-reported memory for abuse depends upon victim-perpetrator relationship. J. Trauma Dissociation.

